# Extract from T. L. Papin's Letter

**Published:** 1849-01

**Authors:** 


The following interesting extract is taken from a letter of T. L. Papin, M. D., one of the élèves of the University of St. Louis, now in Paris, to Dr. Pope.

"In the dressings made by M. Boudens, simple cerate and poultices are as little used as possible, and in their stead, cold water irrigations or direct application of pounded ice in bladders, over the wound, is used. This treatment appears to me the more rational; certainly the result is nearly magical in many cases. One very satisfactory consequence of this dressing is, that you are never bothered by those excessive suppurations which large quantities of charpie and poultices will almost always bring on, and by means of which, the patients life is literally drained off.

Jobert, at the Hospital St. Louis, has a very summary way of stopping excessive suppuration of an amputated limb. He washes the stump as clean as possible, and then cauterizes it very thoroughly and deeply with the solid nitrate of silver. I have seen this used in one case with very satisfactory result. There is a surgeon, (whose name I cannot remember, and who practices in one of the provincial cities,) who uses the actual cautery in these cases, with great success. He published an article on this subject some weeks ago, in one of the Paris medical papers, giving an account of his experience in this matter, and calling on the surgeons of Paris to try it; but as he belongs to the medical school of Montpelier, of course no surgeon of Paris will try it for fear that it might succeed. By-the-way, this same actual cautery is very much and very successfully used by Jobert and Gendrin in ulceration of the neck of the womb.

I have seen a great deal of that dreadful complication of wounds: I mean traumatic erysipelas. Blandin and Jobert seem to succeed best in combating this very frequent and very fatal complication. The first uses leeches in large quantities just around the erysipelatous surface and along the course of the lymphatics which go from the inflamed part to the trunk; and the second dresses the inflamed surface and about an inch beyond it, with a very strong solution or ointment of the nitrate of silver. I may say here what I should have said before:—Baudens seldom has, with his ice and cold water dressings, to combat traumatic erysipelas. As for the solution of sulphate of iron, recommended and used by Velpeau in these cases, I have not seen as good results from it as had been led to expect. It is at best, an harmless remedy, and very objectionable, on account of its spoiling all the patients clothes and bedding, in spite of the precautions which may be taken to prevent it.

One word more, before I close this letter, on the appearance of the holes made by balls from fire-arms in the fleshy parts. Druitt says, (and I believe that many American surgeons teach the same thing,) "that when a musket ball has penetrated a fleshy part, there is seen a hole, perhaps rather smaller than the ball itself, with its edge livid and inverted; and if the ball have passed completely through, there will be another, *larger* and more ragged orifice, with its edge everted." Now, in over a hundred cases which I have noted down, which have been pointed out to the class in my presence by Velpeau, Roux, Blandin, Michou, Laugier, Jobert, Robert, Boyer, and Baudens, have always and invariably seen the following to be the true appearance of both orifices, viz: when a ball had penetrated into a fleshy part, it made a hole fully as large as the ball itself, the edge being smooth and round, and of a livid color; and if the ball had come out, the opening which *it made at coming out was twice and sometimes three times smaller* than the orifice by which it had entered the flesh. The shape of this second orifice was generally ragged, but in several cases, it had the exact appearance of a clean cut, half an inch long made by a surgeon's bistoury; so much so, in fact, that I have heard Blandin, Velpeau, and others, repeatedly ask these patients whether the balls had not been cut out by some one, but the invariable answer was that they had not.

I had the pleasure of meeting Professor Horner, of Philadelphia, in M. Roux's service at Hotel Dieu, and had a conversation with him on this subject, after having pointed out to him several cases which presented themselves to our notice. He says that this error of the English and American surgeons arose from experiments which were made by Sir Charles Bell, who shot a pistol at a pine board, and on examining the board, he found that the orifice of entry was indeed much smaller than the other, and hence the conclusion that it must be the same thing when a ball passes through human flesh, instead of a pine board.

I fear that this long letter will prove a bore to you, my dear Doctor, but you asked me for something new in surgery, and I send you what has interested me the most, hoping that it may prove of some interest to you, also."—*St. Louis. Med. and Surg. Jour.*

